# Ergonomic Issues in Brain-Computer Interface Technologies: Current Status, Challenges, and Future Direction

**DOI:** 10.1155/2020/4876397

**Published:** 2020-02-07

**Authors:** Hyun Jae Baek, Hyun Seok Kim, Minkyu Ahn, Hohyun Cho, Sangtae Ahn

**Affiliations:** ^1^Department of Medical and Mechatronics Engineering, Soonchunhyang University, Asan, Republic of Korea; ^2^Institute for Brain and Cognitive Engineering, Korea University, Seoul 02841, Republic of Korea; ^3^School of Computer Science and Electrical Engineering, Handong Global University, Pohang, Republic of Korea; ^4^National Center for Adaptive Neurotechnologies, Department of Neuroscience and Experimental Therapeutics, Albany Medical College, Albany, NY, USA; ^5^Department of Psychiatry, The University of North Carolina at Chapel Hill, Chapel Hill, NC, USA

The brain-computer interface (BCI) is defined as a computer technology that can interact with the nervous system by decoding information from neural activity. It is an increasingly becoming reliable piece of technology that involves the brain to sense, analyze, and translate signals into commands for the purpose of providing a human computer interaction that will change our life, particularly of patients who suffer from paralysis or similar neurological conditions. BCI enables the communication with computers and controls of external devices such as robotic agents.

Since BCI research emerged a few decades ago, it has mainly focused on signal processing methods to increase its accuracy for its applications such as virtual keyboards, wheelchair controls, or rehabilitation training. BCI is now ematuring towards being more realistic and practically plausible; therefore, it is time to take ergonomics (or human factors) into account as part of the BCI design processes. In addition to improving BCI system performance such as accuracy, brain signal measurements should be more convenient and easier, and the BCI paradigm including audio or visual stimulation should not make the user tired.

The goal of this special issue is to share cutting-edge research and applications on user-friendly BCI solutions such as new concept paradigms, innovative sensors, devices, and signal-processing algorithms. To achieve this goal, the editorial team focused on the core technologies that could contribute to the implementation of possible future BCI solutions and identified the nine representative manuscripts submitted to the special issue.

This special issue includes 2 review papers and 7 research papers on state-of-the-art BCI technologies that are being studied and developed for enhancing user acceptance. In the review article entitled “Enhancing the Usability of Brain-Computer Interface Systems,” H. J. Baek et al. investigated the EEG electrode technologies for wearable measurements. They also showed new paradigms for minimizing visual or auditory fatigue in BCI process. In the review article entitled “Advances in Hybrid Brain-Computer Interfaces: Principles, Design, and Applications,” Z. Li et al. discussed the research progress of hybrid BCI to improve BCI performance and achieve multifunctional control. In the article entitled “Evaluating a Semiautonomous Brain-Computer Interface Based on Conformal Geometric Algebra and Artificial Vision,” M. A. Ramírez-Moreno and D. Gutiérrez proposed a semiautonomous approach based on a conformal geometric algebra model that solves the inverse kinematics of the robot on the fly to control a robotic arm with less mental fatigue. In the article entitled “Driving Fatigue Detection from EEG Using a Modified PCANet Method,” Y. Ma et al. presented a novel feature extraction strategy developed by integrating the principle component analysis and a deep learning model to achieve high classification accuracy and efficiency in using EEG for driving fatigue detection. In the article entitled “Covert Intention to Answer “Yes” or “No” Can Be Decoded from Single-Trial Electroencephalograms (EEGs),” J. W. Choi and K. H. Kim showed possibility to decode binary intentions from multichannel single-trial EEGs while covertly answering to self-referential questions with either “yes” or “no.” In the article entitled “Comparison of Visual Stimuli for Steady-State Visual Evoked Potential-Based Brain-Computer Interfaces in Virtual Reality Environment in terms of Classification Accuracy and Visual Comfort,” K. Choi et al. described the importance of an appropriate visual stimulus to enhance the overall performance of the steady-state visual evoked potential-based BCIs in virtual reality (VR) environments. In the article entitled “Impact of Speller Size on a Visual P300 Brain-Computer Interface (BCI) System under Two Conditions of Constraint for Eye Movement,” R. Ron-Angevin et al. investigated the effect of speller size on P300-based BCI usability, measured in terms of effectiveness, efficiency, and satisfaction under overt and covert attention conditions. In the article entitled “Application of Deep Learning in Neuroradiology: Brain Haemorrhage Classification Using Transfer Learning,” A. M. Dawud et al. proposed deep learning approach to solve the problem of identifying brain haemorrhage from brain computer tomography images. In the article entitled “An Optimized Channel Selection Method Based on Multifrequency CSP-Rank for Motor Imagery-Based BCI System,” J. K. Feng et al. proposed a common spatial pattern rank channel selection method for multifrequency band EEG to prevent deteriorated BCI performance by redundant information.

